# Mapping and Handling Conflicts of Interest in Deceased Organ Donation: How to Handle Ethical Issues and Build Trust in the Healthcare Team

**DOI:** 10.3389/ti.2025.14235

**Published:** 2025-10-06

**Authors:** David Shaw, Nichon Esther Jansen, Alicia Pérez-Blanco, Anne Floden, Rutger Jan Ploeg, Jessie Cooper, Tineke Jentina Wind, Dale Gardiner

**Affiliations:** ^1^ Institute for Biomedical Ethics, University of Basel, Basel, Switzerland; ^2^ Care and Public Health Research Institute, Maastricht University, Maastricht, Netherlands; ^3^ Dutch Transplant Foundation, Leiden, Netherlands; ^4^ Organización Nacional de Trasplantes, Madrid, Spain; ^5^ Södra Älvsborg Hospital, Borås, Sweden; ^6^ Institute of Health and Care Sciences, The Sahlgrenska Academy, University of Gothenburg, Gothenburg, Västergötland, Sweden; ^7^ Oxford Transplant Centre, Churchill Hospital, Oxford University Hospitals NHS Trust, Oxford, United Kingdom; ^8^ School of Health Sciences, City University of London, London, United Kingdom; ^9^ Maastricht University Medical Centre, Maastricht, Limburg, Netherlands; ^10^ Nottingham University Hospitals NHS Trust, Nottingham, United Kingdom

**Keywords:** ethics, conflicts, organ donation, policy, law

## Abstract

It has been suggested that there is a significant conflict of interest between providing best care for the dying patient and a subsidiary role in facilitating the donation process. Should healthcare professionals who are involved in a patient’s care and determination of death also be involved in discussing donation with families? If they are involved, should they disclose this potential conflict of interest? In this paper we address the issue of conflicts of interest in organ donation by examining current best practice in four European countries (Sweden, Netherlands, the United Kingdom and Spain) and discuss whether having clear separation of roles in order to avoid conflicts is preferable to having the same physician (or team) handle both the dying process and donation. We also analyse the benefits and burdens of disclosing such potential conflicts.

## Introduction

A conflict of interest is any motivation or circumstance that might bias a professional’s decision-making in a particular situation. Perhaps the classic example is where a researcher receives substantial payment linked to results from a pharmaceutical company whilst conducting a clinical trial involving their product: the financial contribution can constitute a significant conflict of interest that could bias the objectivity of the research endeavour.

At first glance, deceased organ donation might not seem like a particularly likely context for conflicts of interest in staff caring for a potential organ donor; as they often see no benefit to themselves or the patients under their direct care from any subsequent transplant. However conflicts or tensions in role responsibilities can arise between the different professional duties of healthcare professionals.

In normal medical practice, if a patient is ill, the doctor’s job is to help them get better; if a patient is dying and can no longer be saved, it is the doctor’s job to reduce their suffering; if a patient wants to become an organ donor at the end of life, it is the doctor’s job to facilitate that goal wherever possible–this is regarded as a normal part of end-of-life care in many countries [1, 2]. However, a potential conflict of interest can arise if the same healthcare professional is deciding that a patient can no longer be saved, determining that a patient will die, and also raising the issue of organ donation with the patient’s family. If the doctor is perceived as “pushing” donation, whatever their motivations, it might appear that he or she might not have made the maximum possible effort to save the patient’s life. Some fear that this is particularly the case with donation after circulatory determination of death (DCD), where there are less formal criteria (compared to neurological criteria for death) for deciding to instigate end-of-life care and remove mechanical ventilation [3]. While this fear may only represent the risk of a *perceived* potential conflict of interest, rather than a genuine conflict of interest, it would be unfortunate if any family ever got this impression.

In a position paper issued by various American medical associations, it is stated that “If real or perceived conflicts arise between the goals of providing optimal end-of-life care and the goals of procuring organs, delivery of quality end-of-life care should take priority.” [4] It is true that end-of-life care should not be subordinated to “organ procurement” (itself an unfortunate, transactional term that betrays other biases; for which we will use the term “recovering”), but it can also be argued that organ donation is a key part of end-of-life care. Particularly if a patient has made clear that they want to be a donor and save lives. It is this very fact–that organ donation is so closely linked to, and is sometimes part of, end-of-life care and that recipients are in desperate need for a lifesaving transplant–that makes conflicts of interest an important ethical issue.

As authors, we reject the claim that organ donation is in competition with optimal end-of-life care, and thus that a conflict always exists between the duty to preserve life and reduce suffering, and the possibility of organ donation. We consider, like others, that organ donation can and should be an integral part or end-of-life care for patients who have an autonomous wish to become donors. Facilitating organ donation does not mean that the care given to the patient will in any way be sub-optimal, indeed there is much to suggest it will be superior [5].

The question this paper explores concerns conflicts of interest related to role responsibility and disclosure. Given the risk of conflicts of interest, real or perceived, should it be the same healthcare professional or team who handles the decision that further treatment is futile *and* the initiation of the organ donation process and dialogue with the family? The stakes are high for everyone involved. Dying patients, their families, recipients, transplant teams are at the sharp end of these conflicts can be those caring for a potential donor.

Different countries and donation systems have different approaches to this issue.

## The Organ Donation Family Approach: Four European Examples

To inform our analysis of conflicts of interest, it will be helpful to describe how the donation process unfolds and interacts with other aspects of end-of-life care in different countries: Sweden, the Netherlands, the United Kingdom and Spain (all four of which have both DBD and DCD donation). Each of these countries has a different process, with some favouring greater separation of roles than the others. The descriptions here are based on the expertise of the authors of this paper in clinical practice in their respective countries, and their knowledge of the applicable guidance. From the outset, it should be noted that the end of life and organ donation healthcare team involves not only doctors, but a multi-disciplinary team. For readability, we will often shorthand the multi-disciplinary team to doctors, who in the countries we outline, are typically the senior healthcare decision maker.

In Sweden, the conversation about donation is normally initiated by the intensive care unit (ICU) doctor and ICU nurse, who should also be available to discuss the topic later in the process and answer any questions that the family might have [6]. In some regions of Sweden a donation specialist nurse (DOSS) is also involved. The organ donor register is consulted after a “breakpoint decision” (that further treatment is futile) or after the declaration of death. The decisions about end-of-life care as well as donation are made by the intensivists [7]. It is not considered to be a conflict of interest that the treating doctor in the ICU raises the issue of organ donation with the family. Instead, it is seen as one conversation among others with families. The perception from intensivists (ICU doctors) is that it would be a “betrayal” to hand over this conversation to someone else because of the existing bond of trust between intensivist and family; it might arouse suspicion on the part of the family if the doctor they have collaborated with suddenly distances him/herself in these scenarios where organ donation becomes a possibility.

In Sweden, ICOD refers to organ-preserving treatment initiated outside the ICU after an end-of-life decision, solely to assess the possibility of organ donation. Swedish legislation formally recognizes these measures as *organ-preserving treatment*. Following changes to the Transplantation Act 1995:831 in 2022, such treatment may not only continue after it is clear the patient will not survive but may also begin specifically to evaluate donation potential [8]. An end-of-life decision (“breakpoint decision”) must be made by two licensed physicians and documented according to national regulations. Once this decision is made, care transitions to palliative care and organ-preserving treatment, which may include interventions like intubation—even before the patient’s wish to donate are known. These preferences should be clarified as soon as possible, with respect to the next of kin.

Healthcare providers are required to promote organ donation and identify potential donors, even outside the ICU. If a possible donor is found, an ICU physician must assess donation feasibility, often in consultation with a transplant coordinator in organ donation.

Organ-preserving treatment for donation assessment is mainly considered for patients with severe acute brain injuries, where total brain infarction is expected within a short time. Treatment may be provided for up to 72 h after the end-of-life decision.

In the Netherlands there is a strict separation between medical professionals involved in organ donation or in transplantation [9]. This separation also extends to decisions about continuing or ending treatment. It is unlawful to continue with medical treatment after further treatment has been deemed futile. After it is decided that further treatment is futile, typically this is before death determination even in donation after brain death (DBD), the ICU doctor and ICU nurse will (as in Sweden) approach the donor family for organ donation, after consultation of the Donor Register. If consent is given, by the donor via the Register or the family, the organ donor coordinator (ODC) will come to the hospital to organize the donor procedure. In this sense, there is a potential conflict of interest, because the treating physician is also the one who will inform the family about the patient’s imminent death and initiate the conversation before donation. Nonetheless, many doctors see it as their role to discuss donation with the family. Additionally, in cases where the intensive care doctor works in a transplant center they will have potential or current recipients of organs on their ward, so discussing donation with the family is sometimes regarded as a particular conflict of interest. However, in the Netherlands the law on organ donation strictly separates roles after consent: “Before an organ is removed, death is determined by a doctor who may not be involved in the removal or implantation of the organ.”

In 2022–23 the Netherlands ran a one-year pilot during which the treating physician was asked to have early contact with the ODC, before the bad news was shared with the family. In practice, this meant that as soon as the treating physician consulted the Donor Register, the ODC was requested to get in contact with the doctor. During this first moment of contact the ODC gave information using a checklist on four topics; medical suitability for donation, legal framework, planning and logistics, and preparing the donor conversation. This last topic included the possibility for the physician to invite the ODC to approach the donor family together. This collaborative approach was not performed very often, as there was some hesitation from both the doctors and the ODC’s. The few times an ODC was involved in the donation conversation, this was perceived positively by the physician. One of the reasons for this was that a collaborative approach avoids a potential conflict of interest, as perceived by the doctor: the division of roles means that the ODC could be the one who raised donation while the physician could concentrate on the end-of-life care decision.

In the Netherlands, ICOD started with a pilot study in 6 hospitals in 2018; after the success of this study the protocol was implemented nationally according as described here:

“The roles of the emergency physician, neurosurgeon, and neurologist were clearly defined and entailed the identification of potential organ donors within their patients with acute brain injury that had a futile prognosis. These physicians then had to consult the Donor Registry (DR) after identification of a potential organ donor in the ED. Once a patient met the criteria, and if the intensivist was not already part of the decision-making in the ED, the emergency physician, neurosurgeon, or neurologist would contact the intensivist for consultation about the possibility of organ donation and ICU admission. If family members were present, they would be informed about the futility of treatment by the neurologist, neurosurgeon, and emergency physician. Whether or not organ donation was concurrently discussed in the ED or would be deferred to a later moment (ie, if families were too emotional), was left to the clinical judgment of the physician. As per protocol, the possibility was open to transfer these patients to the ICU in order to give the family more time to grieve, discuss organ donation, and start end-of-life care” [10].

In the United Kingdom two senior doctors must agree on the decision to withdraw life-sustaining treatment before potential organ donation. National Institute for Health and Care Excellence (NICE) guidance states that “a multidisciplinary team (MDT) should be responsible for planning the approach and discussing organ donation with those close to the patient” [11]. It is explicitly stated that the MDT should include a Specialist Nurse for Organ Donation (SNOD). SNODs are employed by NHS Blood and Transplant (NHSBT) but are linked to each ICU or attend on a regional on-call basis. The most recent national guidelines for provision of intensive care services refers to the clinician handing over to the SNOD for the donation conversation:

Where organ donation can potentially be offered for a patient, it would be common for the intensive care consultant, intensive care nurse and SNOD to meet the family together. The consultant would lead on breaking bad news before handing over to the SNOD when it is clear that the family have accepted the inevitability of their loss and are ready to consider what may happen next. Involvement of the SNOD in this way provides timely information and support for the family, and significantly increases the consent rate [12].

A 2022 multi-professional endorsed guidance, known as the Donations Action Framework, states that “The individual leading the approach to the family for organ donation must be suitably trained and qualified, have the time to support the family and have sufficient knowledge and skill to sensitively answer any questions.” The Human Tissue Authority, which regulates organ donation in the UK, is of the opinion that specialist nurses are the most suitable persons to lead a donation discussion with the family, working in collaboration with the treating clinical team [13].

A historic area of concern in UK practice was how to introduce the SNOD into the conversation, potentially before transitioning care to palliation had been raised with the family and certainly before donation had been raised by the healthcare team treating the patient. NHSBT guidance suggests the SNOD is introduced as a “specialist nurse that we work with on the unit and who helps support families at this time.” [14] The UK Donation Ethics Committee considered there is no conflict between early involvement of the SNOD with the treating team or the patient’s family but there would be a conflict of interest if the SNODs were to provide medical care to potential donors whilst they are still alive [15].

The UK does not practice ICOD. Non-therapeutic elective ventilation for the purpose of organ donation is currently considered to be against current national guidance (Reference Donation Actions Framework). In the UK patients who are mechanically ventilated in the emergency department with devastating brain injury are admitted to the ICU for the primary purpose of neuro-prognostication not organ donation, even if organ donation is a likely possibility in time [16].

In Spain, decisions regarding the withdrawal of life-sustaining treatment and end-of-life care are made by a multidisciplinary team, which includes all senior intensivists of the unit where the patient is admitted and other specialists (from other hospital units that have taken part in patient care) at dedicated sessions. In Spain unlike the other countries discussed the donor coordinator DC is often a doctor. If the DC is working as an intensive care doctor in the unit where the patient is admitted, he or she refrains from taking part in the WLST decision. The clinical discussion will be led by another member of the treating team. However, this does not preclude them from performing their duties as intensivists, providing optimal care for their patients. The decision to discuss treatment futility and the possibility of WLST is made collectively by the medical team—including multiple intensivists and specialists from other services—along with the patient’s family. At this stage, the DC remains uninvolved to preserve the integrity of the decision-making process.

These mandates are part of the recommendations issued by the Spanish Intensive care society (SEMICYUC) [17], the national protocol of DCD [18] and the national guidelines on intensive care to facilitate organ donation (ICOD) [19]. The latter scenario is nuanced, as the family approach is made before the patient is dead (early interview). Donor coordinators are specifically trained to request consent for admitting patients that typically have a devastating brain injury and fatal prognosis in ICU to preserve the option of organ donation [20]. The maintenance and assessment may end up with a brain death donation process (DNDD) or a DCD if the patient does not evolve to brain death or the family request to finalise the maintenance at any stage of the admission. If the patient was admitted in the ICU, not to preserve organ donation but to attain curative goals and the multidisciplinary team decides to withdraw treatment, the treating physician will explain the prognosis to the family and share the decision with them. Only when the family agrees upon WLST and the shift from active treatment to palliative care, will the DC approach the family to discuss donation opportunities. Notification/referral of the possible donor to the DC must be done in a timely and early fashion according to national guidance, for the DC to assess medical eligibility and properly plan the family approach. In addition, if the patient is not a medically suitable organ donor, but their family asks for information about organ donation or voices that the patient wished to be an OD, the DC will always have a conversation with them.

In Spain, timing to refer the possible donor to the donor coordinator is more flexible. For example; if the family announces the patient’s wishes to donate, the intensivist will notify the case to the coordinator and subsequently leave them to have a conversation about organ donation options; or if the intensivist wants to consult the coordinator to establish whether the patient’s disease (e.g. patients with a rare disease or history of cancer) means that donation is not feasible. In the latter case, the coordinator will have time to study the disease and learn about the specific evaluation approach. Thus, when the coordinator approaches the family, they can ask about details of the medical history, provide information about the diagnostic test that may be needed to assess each organ suitability or to give good reasons to rule out organ or tissue donation.

The legislation states that healthcare professionals must consult the advanced directives registry and learn from the family if organ donation was consistent with the wishes and values of the person. National recommendations establish that conversations about deceased organ donation should always be led by the DC (regulation established in the national donation protocol) [15]. Key to the Spanish approach is the fundamental point that intensivists will spell out the patient’s prognosis and the treatment options to the family but the option of organ donation is usually presented by the donor coordinator.

## Ethical Analysis

### Separation of Roles or Continuity of Care?

As is clear from these descriptions from practice in the different countries, there are a variety of approaches to the family conversation donation process, and thus different attitudes regarding the importance of potential conflicts of interest in this context. In Sweden, it is simply not seen as a conflict of interest for the treating healthcare team to also be involved in donation; indeed, it would seem odd or even unethical for them not to be involved. In the Netherlands, there is a potential perceived conflict but it is standard for the treating doctor to discuss donation with the family before handing over to an organ donor coordinator (though many physicians regard it as their role to discuss donation and are not keen to hand over). In the United Kingdom, there has emerged over time a relatively clear separation of roles. Working collaboratively, the treating doctors will discuss the determination of death or end of life decision with the family with the specialist nurse present; the nurse then handles the donation conversation. Finally, in Spain the treating team is not involved in presenting the option of organ donation but another intensive care doctor, who is the donor coordinator, will raise the topic of donation.

Another important factor to consider is the system of consent for donation that is in place and how that relates to potential conflicts of interest. All of our example countries have opt-out systems in place. It might be assumed that lesser separation of roles would make sense in jurisdictions that have adopted a presumed consent (opt-out) system, where the default is that donation is desired unless an objection is registered. One of the purported positives of presumed consent or opt-out systems is that they normalise donation and make it a normal part of end-of-life care rather than a special, out-of-the-ordinary event that necessitates special treatment. If donation is in this way more of a routine part of end-of-life care it would indeed seem strange to delegate it to somebody else rather than having the intensivist also handle the donation discussion with the family. However, while that logic might seem superficially appealing, we should bear in mind the core potential conflict of interest that concerns us here: the role responsibilities and disclosure between the doctors and nurses providing the best possible care to the patient in order to save his or her life, and discussing organ donation with a dying patient’s family. It is precisely *because* presumed consent can be seen as normalising donation to some extent that greater care may need to be taken within healthcare systems that have implemented this consent modality. If donation becomes seen as routine, that might make it more, rather than less likely that a conflict could be perceived as arising between donation and providing the best possible care for the patient.

### The Importance of Trust in the Team

In fact, this initial analysis reveals that providing an objective, overall ethical analysis of conflicts of interest in this context is very difficult due to the socially and institutionally embedded nature of the ethical norms in each of the different countries. While it might be concluded in the abstract that conflicts of interest should always be avoided via a rigid separation of roles, or that continuity of care including donation is more important in all cases, that would be to ignore the particularities of each individual jurisdiction, health service and institution.

Given that healthcare professionals work within legal, ethical and professional frameworks’ and are subject to numerous procedures designed to prevent any conflict affecting their clinical decision-making, the key point here concerns perception. If a conflict of interest is perceived by families, that could call into question (from their perspective) the integrity of the end-of-life care (and organ donation) process. But *whether* a conflict of interest is likely to be perceived depends entirely on the particular institutional and team set-up. If a family feels at ease with their relative’s clinical care, they are unlikely to be uncomfortable either with a new person discussing donation or with the same person doing so [21].

In this sense, potential conflicts of interests are best handled by promoting trustworthiness in the healthcare team and system for patients and their families. Trust is recognised as being extremely important to organ donation processes [22]. If an institution and healthcare team are trusted because they have rigorous, trustworthy systems in place, it will make little difference whether the same person or a different one conducts the donation conversation. This reliance on trust might be taken as pointing towards maintaining continuity of care with the same person starting the donation discussion, because a relationship of trust already exists in that context, and in some hospitals and countries that might well be the case. However, it may be that wider trust in the entire healthcare team and institution is at least as valuable, or even more so. Handing over the ‘baton of trust’ between sequential members of the healthcare team, including to the SNOD, is a frequent metaphor used in NHSBT education and training. In some healthcare systems, trust may best be built and maintained by clear separation of roles; in others, trust is best served by continuity of end-of-life care, including organ donation, and in other systems a hybrid of both.

### Donation Physicians–A Special Case

In the Netherlands, Sweden and the UK some doctors have dual paid roles as both donation physician and intensive care doctor. In these countries the donation physician role is typically involved responsibility for strategic leadership and management in championing donation rather than direct donor coordination of an actual donor’s care. In Spain the donation physician is the donor coordinator but not at the same moment in time. The UK Donation Actions Framework states that “the role of [donation physicians] is a managerial rather than a clinical responsibility… should not be considered, simply by nature of their role, to have any specific conflict of interest.” [23].

Even so, can the desire to promote donation by these employed donation physicians be a conflict of interest that should be disclosed to the family when the doctor is leading end-of-life care in their capacity as the duty intensive care doctor for patient treatment that day.

Again, at first glance, disclosure seems a robust mechanism that minimises and makes transparent potential conflicts of interest. However, disclosure puts the onus on judging the importance of the information disclosed, onto the receiver. In donation approaches it asks the family to determine whether a role disclosure represents a conflict of interest and whether they should be concerned about this [24]. Given the grief and stress the family is already under, this is likely to be counterproductive, and risks increasing mistrust. Declaring a conflict where none was previously perceived is likely to confuse families and patients, as recognised by the Canadian Medical Association’s “Ethics Guide Recommendations for Organ-Donation–Focused Physicians”:

Disclosure is context specific and depends on the donation physician's role, the circumstances, and the relationship with the patient and family. Disclosure is not necessary if it has no bearing on the situation or the relationship with the family. If the physician, as most responsible physician (MRP), has been treating the patient, he/she should disclose his/her role as a donation physician once donation conversations begin with the family. The disclosure should be made regardless of whether the donation physician role is clinical or administrative [25].

There is also potential for inter-professional issues regarding conflicts of interest. This is because some staff might see a conflict of interest between a doctor’s dual role in providing end-of-life care and being a donation physician. In terms of the family, the most important aspect of any such issues is that they are resolved in a way that does not without-merit lessen family trust in the process. Relatedly, some members of staff might themselves have a personal conflict with particular aspects of care, such as donation after circulatory determination of death; in such cases, the staff member can invoke conscientious objection to avoid participation [26].

The Canadian guidance also mentions that “Donation physicians should build institutional trust by openly engaging with staff about their role” – but we would suggest that building institutional trust–and particularly “trust in the team” - among families of potential donors (and patients generally) is even more important. Trust can be built in different ways, but fundamentally important for trust of the family is that the professional making the request is well trained, open to family questions and good at communication.

### Other Potential Conflicts

An important but more specific conflict concerns the timing of disclosure to the family that donation might be a possibility, in relation to when a donation coordinator is notified. The key issue is whether to notify coordinators of the potential for donation before discussing with family. It could be perceived as a potential conflict of interest if the first step towards donation is taken without first consulting the family (even if there is not really a conflict because the decision to stop treatment was already made independently). Again, local practices on this issue vary. The UK Donation Ethics Committee took a strong position on this topic, stating that:

Contact between the clinical team treating the potential donor and the SN- OD before the decision has been made to withdraw life-sustaining treatment is ethically acceptable. Advantages include identifying patients who are not suitable donors, and avoiding distressing delays to the family if the SN-OD has to travel some distance to get to the unit [13].

Thus, the protection for the patient is that the decision for withdrawal of life-sustaining treatment is independent of the SNOD or considerations of organ donation.

Another potential clinical conflict of interest occurs in occasional cases where the potential recipient of an organ is on the same ward as a potential donor. This is a common occurrence in heart and liver transplant units and particularly in paediatrics, where the sickest children in the country are often cohorted in the largest paediatric intensive care units, typically which are also paediatric transplant centres. Independent and transparent allocation rules are vital to minimise either a real or perceived conflict of interest. While it does not change the nature of the conflict between doing one’s best for the dying patient and seeking to facilitate donation, this reduction of moral distance from the recipient could make more members of staff subject to potential conflicts. (It should also be considered whether the degree of trust that families will have in healthcare systems and institutions could also be affected by whether they are public or private. It is possible that the potential for conflicts of interest to arise is likely to be higher in private, for-profit institutions.)

Finally, it should also be acknowledged that family member’s decision-making can itself be affected by quite severe conflicts of interest. The classic example of this is the “family overrule” or override where the patient is a registered organ donor and one or more family members want to prevent donation. Here, the conflict is an emotional one: they have just lost a loved one and their emotions are in conflict with the wish to respect the dying wish of the patient. Healthcare professionals can help resolve this conflict with careful counselling and discussion [27].

## Conclusion

It is clear from the description of how donation is handled in four different countries and from the ethical analysis above that there is no universal prescription for how best to handle the donation process with respect to role responsibilities. Insisting on separation of roles could lead to discontinuity of care for families who prefer the same trusted healthcare professional to be involved; equally, some families might be distrustful if they perceive an interest in donation potentially compromising end-of-life care. Instead of seeking a “golden bullet” solution–like disclosure, it will be more productive for healthcare professionals to recognise that the essence of managing conflicts of interest in donation is to build and maintain trust in the healthcare team and system among patients and their families.
